# Measurement of the Psychosocial Work Environment in Spanish: Validation of the Psychosocial Factors Questionnaire 75 (PSF-Q75) to Capture Demands and Resources at Different Levels of Analysis

**DOI:** 10.3389/fpsyg.2020.580196

**Published:** 2020-12-17

**Authors:** Hector P. Madrid, Cristian A. Vasquez, Malcolm Patterson

**Affiliations:** ^1^School of Management, Pontificia Universidad Católica de Chile, Santiago, Chile; ^2^Alliance Business School, University of Manchester, Manchester, United Kingdom; ^3^Institute of Work Psychology, Management School, University of Sheffield, Sheffield, United Kingdom

**Keywords:** psychosocial risks, job demands–resources model, multilevel research, affect at work, stress, engagement

## Abstract

The psychological work environment is composed of both stressful and motivational work conditions at different levels of analysis. However, most relevant theory and research lack an integrative conceptualization and appropriate instrumentation to account for this work context structure. These limitations are particularly present in non-mainstream populations, such as the Spanish community of researchers and practitioners. In this study, based on the job demands–resources model, we present an updated conceptualization in which stressful and motivational psychosocial factors are integrated and defined at the job, the group, and the organizational level of analysis into a single conceptualization. Furthermore, derived from this conceptualization, we present a study of the development and validation of a questionnaire to account for the psychosocial work environment in Spanish, labeled Psychosocial Factors Questionnaire 75 (PSF-Q75), which provides measures for 23 different psychosocial factors. The results of this study supported the questionnaire’s construct, convergent, divergent, and predictive validity, together with its reliability. Thus, this conceptualization and questionnaire provides researchers and partitioners with a more comprehensive approach to the assessment of the psychosocial work environment and promises benefits for interventions in the workplace.

## Introduction

The psychosocial work environment refers to the set of work conditions under which employees perform their activities in organizations (ILO, 1986). The components of this environment are important to be identified and managed because they impact on the experience of health and well-being of employees. As such, traditionally, the psychosocial context at work has been described in terms of stressful conditions, also known as psychosocial risks, that have the potential to impair employee mental health (Leka et al., 2003; Gonzalez-Mule and Kim, 2019). This is the case of, for example, role ambiguity, time pressure, and workload (Karasek, 1979). Alternative models have also included in the definition of the psychological work environment conditions associated with the experience of motivation, such as the case of job autonomy, feedback, skill variety and task significance (Hackman and Oldham, 1976; Oldham and Fried, 2016).

Despite the advance of these issues in the field of organizational and occupational health psychology research and practice (Tetrick and Winslow, 2015; Bakker and Demerouti, 2016; Parker et al., 2017), the theoretical development of stressful and motivational conditions of the psychological work environment has tended to be separated, with some models focused on stress and others on motivation (Kristensen et al., 2005; Morgeson and Humphrey, 2006; Leka and Cox, 2010; Pejtersen et al., 2010). This limits achieving a more comprehensive understanding of psychosocial factors at work. Furthermore, even when the work environment is described by conditions located at different levels of analysis, namely, the job, the group, and the organization as a whole (Bakker and Demerouti, 2018), most theoretical models of psychosocial factors have overlooked this multilevel structure. This constrains the ecological validity of such models. In practical terms, the above limitations also lead to issues about the methodologies to account for the psychosocial work environment in organizations, such as the availability of appropriate instruments to capture both stressful and motivational conditions at different levels of analysis. From a practical view, suitable measurement instruments are essential for diagnosing work conditions in organizations to inform interventions. These issues affect research at international levels as a whole, but they are particularly sensitive to non-mainstream research communities. This is the case for the Spanish population, which is the second widest spoken language in the world with more than three hundred and thirty million speakers in forty-four countries distributed in, for example, Spain, Latin America, and the United States (Lewis, 2009). Thus, the dearth of more advanced methodologies to capture psychosocial factors at work in this cultural setting is an important omission in research and practice.

To address the above limitations in research on the psychosocial work environment, the aim of this study is, based on a multilevel conceptualization of stressful and motivational work conditions, developing and validating a questionnaire in Spanish to measure psychosocial factors at work in organizations. To achieve this goal, we rely on the basics of the job demands–resources model of stress and motivation (Bakker and Demerouti, 2016). Thus, we contribute to organizational and occupational health psychology by describing an updated and integrative conceptualization of work conditions that influences employee well-being and delivering a questionnaire aligned with this conceptualization for the Spanish community of researchers and practitioners.

### Theoretical Framework

To account for both the stressful and motivating work conditions of the psychosocial work environment, we adopted the job demands–resources model proposed in the occupational health psychology literature (Bakker and Demerouti, 2016). According to this model, demands are defined as elements of the work context associated with the experience of stress, which, therefore, may dampen work performance and impair well-being. In turn, resources are the conditions of the work environment that have the potential of motivating employees and therefore facilitating their performance and enhancing their sense of well-being. This distinction implies that demands denote threats for the work and the self, whereas resources entail potential rewards for the same outcomes. Thus, for example, workload and time pressure are primarily threats for work performance, which are prompted by the experience of stress. In turn, resources, such as job control and social support, entail opportunities to do the work beyond its minimum requirements, which is conveyed by the experience of motivation. Thus, demands and resources are not the opposite ends of the same continuum, but independent factors with different meaning and psychological consequences, such that demands are primarily predictors of stress experiences, whereas resources of motivation states (Schaufeli and Bakker, 2004; Schaufeli and Taris, 2014; Lesener et al., 2019).

Furthermore, the psychosocial work environment expressed in demands and resources is given in a multilevel structure. The latter means that elements of the work context are more proximal or distal to the individual employee experience, expressed in the job, the group, and the organization referents (Bakker and Demerouti, 2018). Thus, this multilevel structure defines a hierarchical system in which the diverse psychosocial factors are located (Martin et al., 2016). The job-level of analysis involves those demands and resources that are part of the individual environment of employees, such as workload, time pressures, autonomy, and skill variety (Hackman and Oldham, 1976; Karasek, 1979). In turn, the group-level environment refers to those psychosocial factors with social and interpersonal meaning, which influence all the members of the same work unit or teams, such as the case of conflict and supportive supervision (Ganster et al., 1986; Jehn and Bendersky, 2003). Finally, the organizational-level context is given by distal work conditions affecting all organizational members, independent of their specific jobs and groups. Examples of organizational demands and resources are unfair practices, job insecurity and rewards clarity (Jones and James, 1979; De Witte, 1999; Colquitt and Zipay, 2015).

In the following sections, we present a development and validation study of a questionnaire to measure the psychosocial work environment, based on the above theoretical principles and definitions, distinguishing between demands and resources at different levels of analysis.

## Methods

### Questionnaire Development

In the first stage of the study, we surveyed theoretical developments and empirical research in order to develop an inclusive set of factors describing the psychosocial work environment, examining relevant studies conducted in the fields of the organizational and occupational health psychology. Two graduate students of work and organizational psychology conducted a scoping literature review based on the concepts of “psychosocial work environment,” “psychosocial risks,” “job demands,” and “job resources.” Based on the documents identified, we built an integration of the main demands and resources, organizing them into a single multilevel classification according to our conceptual definitions for the job, group and organizational levels of analysis. This classification served as the theoretical framework for the subsequent development of the questionnaire, which is presented in Table 1.

**TABLE 1:** Conceptual classification of the psychosocial work environment.

**Psychosocial Factor**	**Definition**	**References**
**Job level**		
***Demands***		
Role demands	Lack of clarity about what is expected of the worker and the request of contradictory tasks.	Karasek, 1979
Workload	A large amount of work and number of tasks to be done.	Karasek, 1979
Time pressure	Little time to finish the work, tight deadlines, and fast work pace.	Karasek, 1979
Cognitive demands	Request to paying attention to multiple tasks at the same time, sustained concentration, and memory overload.	Wall et al., 1990
Emotional demands	Request to hide emotions, calm down people and to work on emotionally laden environments.	De Jonge and Dormann, 2003
***Resources***		
Autonomy	Room to make decisions about the way of doing the work, the order and timing to execute the tasks.	Hackman and Oldham, 1976
Feedback	Availability of information about the quantity and quality of work done and job performance.	Hackman and Oldham, 1976
Skill variety	Need to use different and diverse skills and knowledge to do the work.	Hackman and Oldham, 1976
Task significance	Knowledge of how important the job is for other people in the organization, clients, users, and society in general.	Hackman and Oldham, 1976
**Group level**		
***Demands***		
Workload sharing	Unfair distribution of workload, responsibilities, and tasks among workgroup members.	Campion et al., 1993
Conflict	Interpersonal and emotional strain among workgroup members.	Jehn and Bendersky, 2003
Interpersonal violence	Psychological and physical violence, humiliation, and aggression among workgroup members.	Schat and Kelloway, 2005
Autocratic supervision	Authoritarian supervisor behavior expressed in lack of attention of workgroup members’ opinions, ideas, and suggestions.	De Hoogh and Den Hartog, 2009
***Resources***		
Group support	Mutual support and help among workgroup members to do the work.	Campion et al., 1993
Communication	Appropriate information sharing and coordination among workgroup members.	Campion et al., 1993
Participation	Room for giving opinions, making suggestions, and participate in decision making in the workgroup.	Campion et al., 1993
Supervisor support	Supervisor instrumental and emotional supportive behavior to manage the performance and needs of the workgroup members.	Ganster et al., 1986
**Organizational level**		
***Demands***		
Unfairness	An imbalance between effort and rewards.	Colquitt and Rodell, 2015
Politics	Promotion decisions based on favoritism and political behavior rather than merit.	Maslyn and Fedor, 1998
Insecurity	Uncertainty about retaining the job over time.	De Witte, 1999
***Resources***		
Rewards clarity	Clear information about the wage composition and how it is computed.	Jones and James, 1979
Training opportunities	Delivery of training for skill and knowledge development.	Jones and James, 1979
Career development	Opportunities for career opportunities fitting employee interests and goals.	Jones and James, 1979

### Measures

In this stage the questionnaire to measure the demands and resources described in our multilevel classification was built, following the methodology proposed by Hinkin (1995). Thus, the initial step consisted of generating the items for measuring each of the demands and resources described in the classification. Two graduate students of work and organizational psychology independently generated the items. After an initial set of items was produced, the same graduate students, together with the leading authors of this paper, evaluated the items in terms of their content validity, differentiation, overlapping and wording, selecting a final pool of 75 items for further empirical examination. Specifically, at the job level, five scales were developed for role demands, workload, time pressure, cognitive demands, and emotional demands, together with four scales for resources conveyed in autonomy, feedback, skill variety, and task significance. At the group level, four scales for demands were built for workload sharing, conflict, interpersonal violence, and autocratic supervision, together with four scales for the resources of group support, communication, participation, and supervisor support. Finally, at the organization level, three scales were developed for unfairness perceptions, political issues, and job insecurity, together with three scales for the resources of rewards clarity, training opportunities, and career development (Tables 3–5)^1^. The scales consisted of a series of Likert statements for which participants indicate their agreement level, using a 5-point scale (1: Strongly Disagree, 2: Disagree, 3: Neither Agree nor Disagree, 4: Agree, 5: Strongly Agree).

### Data Collection and Sample

We worked with a large food corporation in Latin America to administer the questionnaire to a sample of employees for its subsequent validation. The organization requested the research team to take as little time as possible from employees to answer the questionnaire. Thus, the questionnaire was partitioned in three independent forms, one for each level of analysis of demands and resources. These forms were randomly distributed among the invited participants to avoid response biases linked to systematic nesting of data relative to functional areas and job roles. A sample of 1220 employees, from diverse organizational roles, were invited to participate in the study. Five hundred thirty-four employees responded to the questionnaires (45% response rate), 151 for the job level, 183 for the group level, and 190 for the organization level of analysis. Demographics and organizational information for each sample are summarized in Table 2.

**TABLE 2:** Samples demographics.

**Demographics**	**Sample 1 Job Level**	**Sample 2 Group Level**	**Sample 3 Organization Level**
Gender			
Male	66.9%	68.3%	73.7%
Female	33.1%	31.7%	26.3%
Average age (*SD*)	39.56 (10.12)	40.19 (10.63)	40.99 (10.27)
Educational level			
High School	48.3%	43.7%	48.4%
College	51.7%	56.3%	51.6%
Job role			
Professional and technical staff	42.4%	38.4%	37.4%
Supervisor	45.0%	41.5%	44.2%
Manager	12.6%	16.1%	18.4%
Average organizational tenure (*SD*)	9.70 (9.38)	9.96 (9.01)	10.49 (9.38)

### Data Analysis

The data collected in the study was analyzed with a series of statistical techniques to determine the validity and reliability of the questionnaire. First, confirmatory factor analyses were conducted to examine whether measures of demands and resources, at each level of analysis, fitted the conceptual classification developed (Brown, 2006), which provides information about the construct validity of the questionnaire. Based on the same analyses, convergent and divergent validity between factors of the same models were evaluated using Average Variance Extraction (AVE) (Fornell and Larcker, 1981). Then, in the second round of confirmatory factor analyses, at each level of analysis, additional models were estimated in which first-order latent variables denoting demands were loaded in a second-order latent variable, while first-order latent variables about resources were loaded in an independent second-order latent variable. These models were then compared with models in which both demands and resources were loaded in a single second-order factor. These analyses examined whether demands and resources were independent variables describing the work environment, or if they were components of a single dimension describing general conditions of the psychosocial context. Then, the reliability of measures for each demand and resource was examined using internal consistency analysis, based on the Cronbach’s alpha index (Cronbach, 1951).

Furthermore, criterion-related (predictive) validity analyses of the measures of demands and resources in relation to employee stress and motivation were conducted, using affective measures as indicators of these outcome variables, which were analyzed using structural equation modeling (Kline, 2011). Specifically, we used as dependent variables negative feelings because they are emotional components of stress, and positive feelings since they are rudiments of motivation (Warr, 2007). Affect was measured with 6 items from the scale of Warr et al. (2014). This measure asked participants to report the extent to which they feel in the workplace: enthusiastic, joyful and inspired (positive affect, α = 0.83) and anxious, tense and worried (negative affect, α = 0.74) (1 = never – 5 = Always/Almost always). Specifically, at each level of analysis, measures of negative and positive affect were regressed on the second-order models describing a latent variable for demands and another for resources. In these models, the correlation between the second-order latent variable of demands and resources was fixed to zero, to control issues on non-essential collinearity attributed to common method variance issues due to the use of a cross-sectional design. Thus, the effect estimated between demands and resources relative to negative and positive affect, respectively, was based on non-shared variance between these predictors.

## Results

Results of confirmatory factor analysis for measures about the job level of analysis, based on 9 factors and their 27 items, showed acceptable goodness-of-fit, χ^2^(df) = 479.39(288), CFI = 0.93, RMSEA = 0.07. Also, results of AVE analysis showed acceptable convergent validity for all the factors estimated, with values over 0.50, except for cognitive demands (AVE = 0.43) and emotional demands (AVE = 0.40), with results slightly below this cutoff criterion. Convergent validity was also supported for all the factors estimated, since their AVE values were over their squared pairwise correlations (Fornell and Larcker, 1981). Moreover, results for the model described by the 8 factors, and their 30 respective items, about group-level variables showed acceptable goodness-of-fit, χ^2^(df) = 746.18(377), CFI = 0.93, RMSEA = 0.07. Convergent and discriminant validity was also supported for all the factors of this model, based on AVE values over 0.5 and over the squared pairwise correlations between factors. Furthermore, factor analysis for organizational level variables, based on 6 factors and their 18 items, also showed excellent goodness-of-fit, χ^2^(df) = 203.05(120), CFI = 0.97, RMSEA = 0.06. The results also supported convergent and divergent validity with AVE values over 0.5 and over the squared pairwise correlations between factors of the model.

Then, at the job level of analysis, the model describing second-order latent variables for demands and resources showed a goodness-of-fit slightly below the standard estimation benchmarks, χ^2^(df) = 682.36(314), CFI = 0.87, RMSEA = 0.09, but its fit was substantially better than the model in which both demands and resources were loaded in a single factor, Δχ^2^(df) = 32(1), *p* < 0.01. At the group level of analysis, the model describing demands and resources showed acceptable goodness-of-fit, χ^2^(df) = 873.02(396), CFI = 0.92, RMSEA = 0.08, while excellent goodness-of-fit was supported for the second-order model for the organizational level demands and resources, χ^2^(df) = 215.10(128), CFI = 0.97, RMSEA = 0.06. Therefore, taking the above together, the construct validity, based on these factor analyses results, for the measures and the classification system of demands and resources at each level of analysis was supported (Tables 3–6).

**TABLE 3:** Confirmatory factor analysis for job resources and job demands.

**ITEMS**	**1**	**2**	**3**	**4**	**5**	**6**	**7**	**8**	**9**
Think about **YOUR JOB** and rate your agreement or disagreement with the statements below (1: Strongly Disagree – 5: Strongly Agree):									
**Autonomy**									
1. You can decide the order to do your tasks	0.79								
2. You can decide when you start and finish your tasks	0.89								
3. You can decide the way you do your work	0.78								
**Feedback**									
4. You receive feedback about how you are doing your work		0.94							
5. You receive information about the quality and quantity of your work		0.93							
6. You receive information about your performance at work		0.94							
**Skill Variety**									
7. You have to use a variety of skills and knowledge at work			0.94						
8. You have to use diverse skills to do your work			0.96						
9. You have to apply your diverse knowledge to do your tasks			0.95						
**Task Significance**									
10. You know how important your work is for other people at work				0.86					
11. You know how important your work is for clients/users of the organization				0.95					
12. You are aware of the impact in society of your work				0.77					
**Role Demands**									
13. You barely know what is expected of you at work					0.56				
14. You are asked for conflicting demands at work					0.89				
15. You have to handle incompatible tasks at work					0.84				
**Workload**									
16. You have to do a large amount of work						0.96			
17. You have to do too many things at work						0.93			
18. You have to manage heavy workloads						0.71			
**Time Pressure**									
19. You do not have enough time to finish your work							0.76		
20. You have to deal with too tight deadlines at work							0.78		
21. You have to work at fast-pace							0.84		
**Cognitive Demands**									
22. You have to pay attention to different tasks at the same time								0.79	
23. You have to concentrate all the time to avoid errors								0.63	
24. You have to use your memory a lot								0.59	
**Emotional Demands**									
25. You have to hide your emotions at work									0.69
26. You have to calm down angry or annoyed individuals at work									0.54
27. You have to work in environments where you feel threatened									0.65
**Average Variance Extracted (AVE)**	0.69	0.88	0.91	0.74	0.61	0.75	0.63	0.43	0.63

**TABLE 4:** Confirmatory factor analysis for group resources and group demands.

**ITEMS**	**1**	**2**	**3**	**4**	**5**	**6**	**7**	**8**
Think about **YOUR WORKGROUP** and rate your agreement or disagreement with the statements below (1: Strongly Disagree – 5: Strongly Agree):								
**Group Support**								
28. In my workgroup, we support each other when somebody needs help	0.95							
29. In my workgroup, we help each other	0.97							
30. In my workgroup, we collaborate to solve problems	0.92							
**Communication**								
31. In my workgroup, communication is good		0.93						
32. In my workgroup, information sharing is good		0.94						
33. In my workgroup, coordination is good		0.79						
**Participation**								
34. In my workgroup, there is room to give opinions			0.92					
35. In my workgroup, there is room to make suggestions on the work we do			0.95					
36. In my workgroup, there are opportunities to participate in decision making			0.80					
**Supervisor Support**								
37. My supervisor plans the work well				0.83				
38. My supervisor distributes the workload in a balanced way				0.82				
39. My supervisor gives clear and precise information to get the work done				0.85				
40. My supervisor offers help to solve problems at work				0.81				
41. My supervisor takes into account the needs of employees				0.84				
42. My supervisor gives recognition for the well-done job				0.88				
43. My supervisor advises employees to improve performance				0.78				
44. My supervisor cares about managing conflict at work				0.89				
**Workload Sharing**								
45. In my workgroup, workload distribution is unfair					0.84			
46. In my workgroup, responsibilities distribution is unfair					0.93			
47. In my workgroup, task distribution is unfair					0.96			
**Conflict**								
48. In my workgroup exists conflict between its members						0.93		
49. In my workgroup exists tension in the way that members interact to each other						0.97		
50. In my workgroup, members have negative relationships with each other						0.88		
**Interpersonal Violence**								
51. In my workgroup, some members insult others							0.90	
52. In my workgroup exists humiliation situations toward some of its members							0.90	
53. In my workgroup, there are situations of aggression and physical violence							0.69	
**Authoritarian Supervision**								
54. My supervisor acts in a very authoritarian way								0.78
55. My supervisor does not listen to different opinions								0.90
56. My supervisor does not pay attention to the ideas proposed by my workgroup								0.89
57. My supervisor makes us to understand s/he is the only important person in the workgroup								0.83
**Average Variance Extracted (AVE)**	0.89	0.78	0.78	0.70	0.83	0.86	0.76	0.72

**TABLE 5:** Confirmatory factor analysis for organizational resources and organizational demands.

**ITEMS**	**1**	**2**	**3**	**4**	**5**	**6**
Think about **YOUR ORGANIZATION** and rate your agreement or disagreement with the statements below (1: Strongly Disagree – 5: Strongly Agree):						
**Rewards Clarity**						
58. The organization clearly informs me how much is my salary	0.84					
59. The organization clearly informs me how my wage is calculated	0.87					
60. The organization clearly informs me how will be my salary at the end of the month	0.85					
**Training Opportunities**						
61. The organization offers the necessary training to do the work well		0.86				
62. The organization supports me to apply to training courses		0.94				
63. The organization offers training courses to develop new skills		0.83				
**Career Development**						
64. The organization offers development opportunities that fit my goals			0.91			
65. The organization offers job opportunities of my interest			0.92			
66. The organization offers attractive career opportunities			0.94			
**Unfairness**						
67. In the organization, I give much but receive little				0.87		
68. In the organization, I do not earn the appropriate rewards for my work				0.84		
69. In the organization, I feel unfairly treated				0.68		
**Politics**						
70. In the organization, the progress is achieved due to personal favoritism than merit					0.92	
71. In the organization, it is more important to have good connections than performing well					0.95	
72. In the organization, it is more important to be political savvy than showing good performance					0.80	
**Job Insecurity**						
73. The organization does not offer me job stability						0.92
74. The organization does not provide me job security						0.81
75. The organization does not guarantee I will keep my job for much longer						0.71
**Average Variance Extracted (AVE)**	0.73	0.78	0.86	0.64	0.65	0.79

**TABLE 6:** Means, standard deviations, correlations and reliabilities.

**Job (*N* = 141–151)**	**M**	**SD**	**1**	**2**	**3**	**4**	**5**	**6**	**7**	**8**	**9**
(1) Autonomy	4.11	0.74	**(0.86)**								
(2) Feedback	3.64	1.15	0.46**	**(0.96)**							
(3) Skill variety	4.34	0.68	0.29**	0.35**	**(0.97)**						
(4) Task significance	4.22	0.76	0.39**	0.41**	0.56**	**(0.89)**					
(5) Role demands	2.10	0.96	−0.37**	−0.60**	−0.22**	−0.41**	**(0.80)**				
(6) Workload	3.60	0.84	−0.19*	−0.21*	0.23**	−0.01	0.19*	**(0.89)**			
(7) Time pressure	3.26	0.90	−0.35**	−0.22**	0.12	−0.12	0.16	0.68**	**(0.84)**		
(8) Cognitive demands	3.92	0.69	0.08	0.13	0.49**	0.27**	0.04	0.48**	0.35**	**(0.68)**	
(9) Emotional demands	2.70	0.88	−0.28**	−0.34**	0.00	−0.19*	0.48**	0.29**	0.33**	0.25**	**(0.66)**
**Group (*N* = 174–183)**	**M**	**SD**	**10**	**11**	**12**	**13**	**14**	**15**	**16**	**17**	
(10) Group support	4.36	0.80	**(0.96)**								
(11) Communication	4.08	0.83	0.76**	**(0.91)**							
(12) Participation	4.19	0.84	0.64**	0.68**	**(0.91)**						
(13) Supervisor support	4.02	0.88	0.30**	0.47**	0.52**	**(0.95)**					
(14) Workload sharing	2.32	0.98	−0.47**	−0.54**	−0.53**	−0.50**	**(0.94)**				
(15) Conflict	1.92	0.93	−0.50**	−0.51**	−0.46**	−0.47**	0.56**	**(0.94)**			
(16) Interpersonal violence	1.46	0.77	−0.44**	−0.46**	−0.47**	−0.44**	0.49**	0.60**	**(0.85)**		
(17) Autocratic supervision	1.61	0.89	−0.20**	−0.27**	−0.43**	−0.67**	0.42**	0.54**	0.55**	**(0.91)**	
**Organization (*N* = 182–190)**	**M**	**SD**	**18**	**19**	**20**	**21**	**22**	**23**			
(18) Rewards clarity	4.15	0.93	**(0.88)**								
(19) Training opportunities	3.41	1.11	0.50**	**(0.91)**							
(20) Career development	3.60	1.01	0.46**	0.72**	**(0.95)**						
(21) Unfairness	2.58	0.97	−0.44**	−0.45**	−0.49**	**(0.83)**					
(22) Politics	2.63	1.15	−0.33**	−0.50**	−0.54**	0.54**	**(0.91)**				
(23) Insecurity	1.78	0.86	−0.30**	−0.35**	−0.39**	0.37**	0.35**	**(0.84)**			

Reliabilities are in bold and displayed in parentheses in the diagonal. *p < 0.05, **p < 0.01.

In terms of reliability analysis, 21 scales showed values over α = 0.70, but slightly lower reliabilities were observed for cognitive demands, α = 0.68, and emotional demands, α = 0.66. Therefore, acceptable reliabilities were supported for the scales measuring demands and resources at the job, group and organizational level of analyses.

**FIGURE 1:**
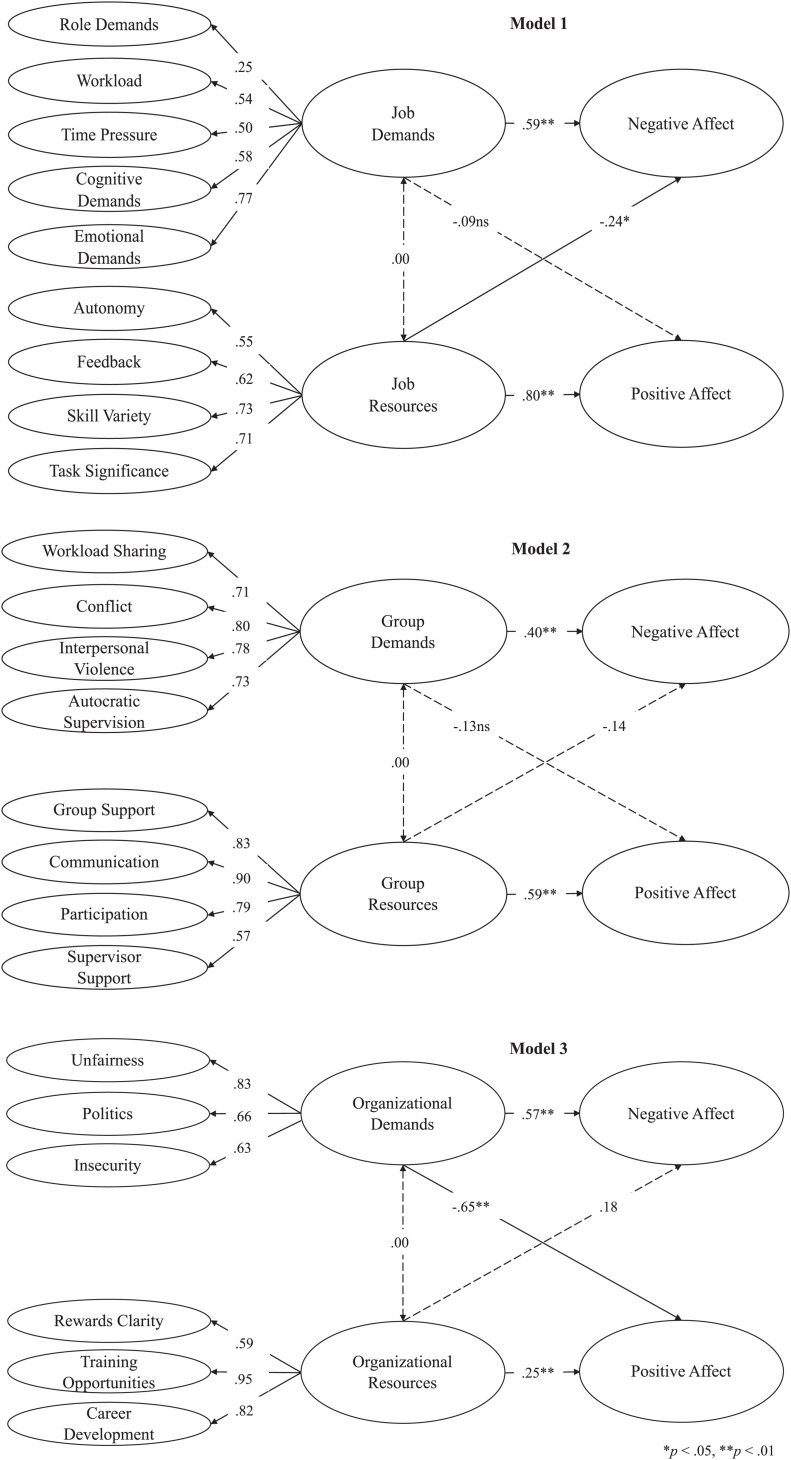
Structural equation models for criterion-related validity.

Finally, criterion-related validity analyses, based on structural equation modeling, showed that job level demands were positively related to negative affect, *b* = 0.59, *SE* = 08, *p* < 0.01, but not related to positive affect, *b* = −0.09, *SE* = 09, *p* > 0.05, whereas job level resources were positively related to positive affect, *b* = 0.80, *SE* = 06, *p* < 0.01, and negatively related to negative affect, *b* = −0.24, *SE* = 09, *p* < 0.05 (Table 7 and Figure 1). At the group level of analysis, demands were positively related to negative affect, *b* = 0.40, *SE* = 10, *p* < 0.01, but not related to positive affect, *b* = −0.13, *SE* = 11, *p* > 0.05, whereas resources were positively related to positive affect, *b* = 0.59, *SE* = 08, *p* < 0.01, but not related to negative affect, *b* = −0.14, *SE* = 11, *p* > 0.05 (Table 8 and Figure 1). Finally, organizational level demands were positively related to negative affect, *b* = 0.57, *SE* = 09, *p* < 0.01, and negatively related to positive affect, *b* = −0.65, *SE* = 09, *p* < 0.01, while organizational level resources were positively related to positive affect, *b* = 0.25, *SE* = 10, *p* < 0.01, but not related to negative affect, *b* = 0.18, *SE* = 09, *p* > 0.05 (Table 9 and Figure 1). These results, in balance, supported the predictive validity of the questionnaire and the theoretical classification of demands and resources underlying it.

**TABLE 7:** Structural equation modeling for job resources and job demands (model 1).

**First-Order Factor**	**Factor Loadings**	**Second-Order Factor**	**Negative Affect**	**Positive Affect**
Role demands	0.25	Job Demands	0.59 (0.08)**	−0.09 (0.09)
Workload	0.54			
Time pressure	0.50			
Cognitive demands	0.58			
Emotional demands	0.77			
Autonomy	0.55	Job Resources	−0.24 (0.09)*	0.80 (0.06)**
Feedback	0.62			
Skill variety	0.73			
Task significance	0.71			

*N* = 151, χ^2^(df) = 911.19 (481), CFI = 0.87, TLI = 0.86, RMSEA = 0.08, SRMR = 0.15. **p < 0.01.

**TABLE 8:** Structural equation modeling for group resources and group demands (model 2).

**First-Order Factor**	**Factor Loadings**	**Second-Order Factor**	**Negative Affect**	**Positive Affect**
Workload sharing	0.71	Group Demands	0.40 (0.10)**	−0.13 (0.11)
Conflict	0.80			
Interpersonal violence	0.78			
Autocratic supervision	0.73			
Group support	0.83	Group Resources	−0.14 (0.11)	0.59 (0.08)**
Communication	0.90			
Participation	0.79			
Supervisor support	0.57			

*N* = 183, χ^2^(df) = 1307.56 (581), CFI = 0.88, TLI = 0.87, RMSEA = 0.08, SRMR = 0.24. **p < 0.01.

**TABLE 9:** Structural equation modeling for organizational resources and organizational demands (model 3).

**First-Order Factor**	**Factor Loadings**	**Second-Order Factor**	**Negative Affect**	**Positive Affect**
Unfairness	0.83	Organizational Demands	0.57 (0.09)**	−0.65 (0.09)**
Politics	0.66			
Insecurity	0.63			
Rewards clarity	0.59	Organizational Resources	0.18 (0.09)	0.25 (0.10)**
Training opportunities	0.95			
Career development	0.82			

*N* = 190, χ^2^(df) = 443.93(241), CFI = 0.93, TLI = 0.93, RMSEA = 0.07, SRMR = 0.20. **p < 0.01.

## Discussion and Conclusion

In this study, we aimed to develop a questionnaire to measure the components of the psychosocial work environment in Spanish, distinguishing between stressful and motivational work conditions at different level of analysis. For this purpose, we integrated a conceptual classification relying on the job demands-resources model, which indicates that the components of the work environment involve conditions that may impair mental health, called job demands, together with contextual conditions associated with the experience of motivation, labeled as job resources. Furthermore, the classification utilized is based on a multilevel structure in which demands and resources are located at the job, group and organizational levels, depending on how proximal they are to the employees’ individual experience. Finally, demands and resources, at each level of analysis, were defined and supported as predictors of employees’ affective experiences, such that the former is associated with negative affect (e.g., anxiety), whereas the latter with positive affect (e.g., enthusiasm). Based on these principles, the 75-item questionnaire developed, called Psychosocial Factors Questionnaire 75 (PSF-Q75), comprises measures to account for the 23 psychosocial factors, and empirical evaluation largely supported its validity and reliability.

This study contributes to organizational and occupational health psychology by presenting an updated integrative conceptualization in which stressful and motivational work conditions are accounted for in a single model. Furthermore, in theoretical terms, the psychosocial factors are explicitly defined at the proper level of analysis of the work environment. Moreover, the significant contribution of this study is the elaboration and development of a questionnaire to measure psychosocial factors, according to the new conceptualization proposed, particularly in the Spanish speaking community of researchers and practitioners. The simple translation of questionnaires available in, for example, the English language (Kristensen et al., 2005; Morgeson and Humphrey, 2006; Leka and Cox, 2010; Pejtersen et al., 2010), would have been a limited strategy, because most of these questionnaires, to the best of our knowledge, are either focused on demands or resources and do not account for the work environment’s multilevel structure in the design of their scales.

There are limitations to be mentioned about the validation study conducted. First, we used a theory-driven strategy to build the questionnaire, which contributes to its content validity. However, the questionnaire was not submitted to the assessment of subject matter experts; thus, future studies in which, for example, content evaluation of practitioners in the field of psychosocial factors management will provide additional information about the questionnaire’s validity. Second, we validated the questionnaire and the model with appropriate sample sizes to conduct the statistical analysis needed, which were also diverse in terms of gender, organizational tenure, functional areas, and job roles. However, we were unable to test metric invariance between groups derived from these composition variables due to insufficient cases for each comparison group. Thus, replication studies with larger and more diverse samples of participants from different organizations and industries will provide a greater generalization of the results observed. Third, we conceptualize demands and resources at the job, the group, and the organization level of analysis, however, data for all these factors were modeled at the individual level of analysis. This strategy was the most appropriate for work conditions defined at the job level, which are essentially part of the individual environment. However, the most suitable way to test group-level factors should be based on samples of working units or teams, while the examination of organizational factors should be done with samples of organizations. This work involves a greater sampling endeavor in terms of time and resources, which future research could conduct to determine the robustness of the validation results presented here. Finally, the test of the criterion-related validity of demands and resources in relation to employee affect relied on data collected from self-reports in a cross-sectional fashion, which might introduce issues of common method variance (Podsakoff et al., 2012). Thus, future studies should utilize multisource and longitudinal designs.

To sum up, we contribute to organizational and occupational health psychology research and practice with the delivery of a Spanish language questionnaire to account for organizational members’ experience of a comprehensive set of factors embedded in the psychosocial work environment. We trust this tool will support further research in this knowledge domain and the management of working conditions associated with well-being at work.

## Data Availability Statement

The raw data supporting the conclusions of this article will be made available by the authors, without undue reservation.

## Ethics Statement

The studies involving human participants were reviewed and approved by Ethics Committee Pontificia Universidad Católica de Chile. The patients/participants provided their written informed consent to participate in this study.

## Author Contributions

All authors listed have made a substantial, direct and intellectual contribution to the work, and approved it for publication.

## Conflict of Interest

The authors declare that the research was conducted in the absence of any commercial or financial relationships that could be construed as a potential conflict of interest.

## Footnotes

1 The original measures were designed in Spanish language, which are available in the Appendix.

## Appendix

### Original Measures in Spanish

PIENSE ACERCA DE SU PUESTO DE TRABAJO y señale su grado de acuerdo o desacuerdo con las siguientes afirmaciones (1: Muy en desacuerdo; 2: En desacuerdo, 3: Ni de acuerdo ni en desacuerdo; 4: de acuerdo; 5: Muy de acuerdo).

### Autonomía

1. Puede decidir el orden en que realiza sus tareas
2. Puede decidir cuando comenzar y finalizar sus tareas
3. Puede decidir la manera en que realiza su trabajo

### Retroalimentación

4. Recibe retroalimentación acerca de como está realizando su trabajo
5. Recibe información sobre la calidad y cantidad del trabajo que realiza
6. Recibe información acerca de su desempeño en el trabajo

### Variedad de las Habilidades

7. Tiene que utilizar distintas habilidades y conocimientos en su trabajo
8. Tiene que usar diversas habilidades para hacer su trabajo
9. Tiene que aplicar sus distintos conocimientos para realizar sus tareas

### Significado de las Tareas

10. Conoce que tan importante es su trabajo para otras personas de la organización
11. Sabe cuán importante es su trabajo para los clientes/usuarios de la organización
12. Tiene claridad del impacto que su trabajo tiene para la sociedad

### Demandas de Rol

13. Tiene poca claridad de qué es lo que se espera de usted en el trabajo
14. En su trabajo le piden cosas contradictorias
15. En su trabajo le piden tareas que son incompatibles entre sí

### Carga de Trabajo

16. Tiene que realizar una gran cantidad de trabajo
17. Tiene que hacer muchas cosas en el trabajo
18. Tiene que hacer una cantidad excesiva de trabajo

### Presión de Tiempo

19. Tiene poco tiempo para finalizar el trabajo
20. Tiene que cumplir con plazos muy ajustados
21. Tiene que trabajar a un ritmo rápido para hacer sus tareas

### Demandas Cognitivas

22. Tiene que estar pendiente de varias tareas a la vez
23. Tiene que estar concentrado todo el tiempo para evitar errores
24. Tiene que utilizar mucho su memoria

### Demandas Emocionales

25. Tiene que evitar mostrar sus emociones
26. Tiene que tranquilizar a personas que están molestas o enojadas
27. Tiene que trabajar en ambientes en los que se siente amenazado

PIENSE ACERCA DE SU GRUPO DE TRABAJO y señale su grado de acuerdo o desacuerdo con las siguientes afirmaciones (1: Muy en desacuerdo; 2: En desacuerdo, 3: Ni de acuerdo ni en desacuerdo; 4: de acuerdo; 5: Muy de acuerdo).

### Apoyo Grupal

28. En mi grupo de trabajo nos apoyamos cuando algún compañero de trabajo solicita ayuda
29. En mi grupo de trabajo nos ayudamos mutuamente entre los compañeros de trabajo
30. En mi grupo de trabajo colaboramos para resolver los problemas

### Comunicación

31. En mi grupo de trabajo existe una buena comunicación
32. En mi grupo de trabajo existe un buen intercambio de información
33. En mi grupo de trabajo existe una buena coordinación

### Participación

34. En mi grupo de trabajo existe el espacio para dar opiniones
35. En mi grupo de trabajo existe el espacio para hacer sugerencias acerca del trabajo que realizamos
36. En mi grupo de trabajo existen oportunidades para participar cuando se toman decisiones

### Supervisión de Apoyo

37. Mi jefe planifica bien el trabajo
38. Mi jefe asigna de forma equilibrada la carga de trabajo
39. Mi jefe entrega información clara y precisa para hacer el trabajo
40. Mi jefe ofrece ayuda para resolver problemas en el trabajo
41. Mi jefe toma en cuenta las necesidades de los trabajadores
42. Mi jefe da reconocimiento por el trabajo bien hecho
43. Mi jefe da consejos para mejorar el desempeño de los trabajadores
44. Mi jefe se preocupa de resolver los conflictos en el trabajo

### Inequidad de las Cargas de Trabajo

45. En mi grupo la distribución de las cargas de trabajo es injusta
46. En mi grupo la distribución de las responsabilidades es injusta
47. En mi grupo la distribución de tareas es injusta

### Conflicto

48. En mi grupo de trabajo existe conflicto entre sus integrantes
49. En mi grupo de trabajo existe mucha fricción en la forma que se relacionan sus integrantes
50. En mi grupo de trabajo los integrantes se llevan mal entre sí

### Violencia

51. En mi grupo de trabajo hay integrantes que insultan a otros
52. En mi grupo de trabajo hay situaciones de humillación hacia algunos de sus integrantes
53. En mi grupo de trabajo hay situaciones de agresión y violencia física

### Supervisión Autocrática

54. Mi jefe actúa de forma muy autoritaria
55. Mi jefe no escucha opiniones distintas a las de él
56. Mi jefe no presta atención a las ideas propuestas por mi grupo de trabajo
57. Mi jefe nos da a entender que él es la única persona importante de mi grupo de trabajo

PIENSE ACERCA DE SU ORGANIZACIÓN y señale su grado de acuerdo o desacuerdo con las siguientes afirmaciones (1: Muy en desacuerdo; 2: En desacuerdo, 3: Ni de acuerdo ni en desacuerdo; 4: de acuerdo; 5: Muy de acuerdo).

### Claridad de las Remuneraciones

58. La organización me informa claramente cuánto es mi sueldo
59. La organización me informa claramente acerca de cómo se calcula mi sueldo
60. La organización me informa claramente cuál va a ser mi sueldo a fin de mes

### Oportunidades de Capacitación

61. La organización ofrece la capacitación necesaria para hacer bien el trabajo
62. La organización me da apoyo para postular a cursos de capacitación
63. La organización ofrece cursos de entrenamiento para desarrollar nuevas habilidades

### Oportunidades de Desarrollo

64. La organización ofrece oportunidades de desarrollo que se ajustan a mis metas
65. La organización ofrece oportunidades laborales que son de mi interés
66. La organización ofrece oportunidades de desarrollo de carrera que son atractivas

### Inequidad

67. En la organización entrego mucho, pero recibo poco
68. En la organización no recibo las recompensas adecuadas por mi trabajo
69. En la organización me siento injustamente tratado

### Política Organizacional

70. En la organización se progresa por favoritismos y no por el mérito
71. En la organización es más importante tener buenos contactos que hacer bien el trabajo
72. En la organización ser “político” es más importante que tener un buen desempeño

### Inseguridad Laboral

73. La organización no me da estabilidad laboral
74. La organización no entrega seguridad laboral
75. La organización no me asegura que conservaré mi trabajo por mucho tiempo más
